# Why are haematopoietic stem cells in the bone marrow: ontology recapitulates phylogeny

**DOI:** 10.1038/s41375-023-01986-x

**Published:** 2023-07-29

**Authors:** Robert Peter Gale, James Welsh, P. Andrew Karam

**Affiliations:** 1 Centre for Haematology, Department of Immunology and Inflammation, Imperial College of Science, Technology and Medicine, London, UK; 2 Loyola University School of Medicine Department of Radiation Oncology, Chicago, IL USA; 3 US Department of Homeland Security, National Urban Security Technology Laboratory, New York City, NY USA

**Keywords:** Biotechnology, Diseases

## Introduction

Life on Earth began in an aquatic environment giving rise to terrestrial forms during the Devonian Period, about 370 million years ago (Ma) following a semi-aquatic interphase. Fossil evidence indicates the transition from fully aquatic fish into fully terrestrial tetrapods occurred as lobe-finned fishes (sarcopterygians) such as *Tiktaalik* and *Panderichthys* and later intermediate forms (*fishapods*) became increasingly more capable of land excursions, ultimately evolving into fully terrestrial vertebrates (e.g. tetrapods *Ichthyostega* and *Acanthostega)*.

In parallel with the evolution from aquatic to terrestrial life, blood cell production migrated from earlier sites in the thorax and abdomen (e.g. blood islands, aortic mesonephros, liver and spleen [in some species]) to the bone marrow cavity.  This  phylogeny  is ecapitulated in human ontogeny.

Herein, we raise the question of what evolutionary force(s) might underlie this translocation and explore the possibility the translocation of haematopoietic stem cells to the bone marrow cavity occurred as an adaptation to higher levels of environmental radiations when vertebrate life lost the protection conferred by water.

## Radiation and radiation shielding

The four primary types of radiation are alpha, beta, gamma and neutron, as well as cosmic ray-induced muons. Of these, alpha particles can penetrate only a few to several microns and beta particles no more than about 1 cm into water or tissue.

Gamma, muon, and neutron radiations are highly penetrating. Gamma and x-ray radiation are attenuated by any matter interposed between the source and receptor with denser materials making a more effective shield. Neutron radiation is best shielded by materials with a high concentration of hydrogen (e.g.) adipose and other soft tissue, reducing the amount of biological damage done to the underlying tissues (e.g. haematopoietic stem cells).

The only natural source of neutron and muon radiations is cosmic radiation which has remained largely unchanged since life first moved to the land [1]. Neutron radiation at sea level accounts for about 8% of exposure to deep tissues including the bone marrow similar to external gamma radiations. Cosmic ray-induced muons contribute a further 42% of environmental penetrating radiation exposure. However, energetic muons interact only weakly with matter and are not attenuated to the same degree as other penetrating radiations by bone and soft tissues. The remaining exposure to penetrating radiation comes from gamma photons from geologic (41%) and biological (8%) sources.

Ultraviolet (UV) radiations, although ionizing, are not deeply penetrating and therefore unlikely to affect haematopoietic cells except in organisms which live relatively high in the water column and which are more or less transparent to UV radiation [2]. We also note the haematopoietic tissues in vertebrate organisms which burrow into the soil, are primarily nocturnal, or are covered with pigmented skin, scales, feathers, carapaces and so forth are unlikely to be affected by UV radiations. Consequently, for the purposes of this discussion, exposure to UV radiation is not an important factor that could affect the location of the haematopoietic cells.

## Radiation exposure to aquatic and terrestrial organisms

Water is an effective shield against alpha, beta, gamma and neutron radiations. For aquatic organisms this exposure is sufficiently low to be disregarded as an evolutionary selective force. Radiation exposure from dissolved radionuclides (e.g. ^40^K) is similarly small because of the relatively low concentration of radioactive atoms in seawater and the attenuation of even high-energy gamma radiation. Consequently, to organisms living more than a few meters deep within the water column the primary source of radiation exposure is internal biologically incorporated radioactivity (from, primarily ^40^K, ^3^H, and ^14^C), receiving about 0.35 and 0.31 millisieverts per year (mSv y^–1^), respectively, with electrons (e.g. beta radiation and Auger electrons) from dissolved radionuclides contributing only minor radiation exposure to the organism’s outermost layers [3].

Exposure to cosmic ray muons is primarily related to solar activity with rare bursts of up to 0.3 mSv from occasional high-energy cosmic events such as supernovae, gamma ray bursts, and/or solar superflares, every few to several million years [4–6]. Organisms living atop of the water column (e.g. plankton) receive additional radiation from cosmic ray neutrons (about 0.5 mSv y^−1^). However, most of these organisms are small and have rudimentary circulatory systems without blood cells.

In summary, radiation exposure to aquatic organisms is dominated by two factors, cosmic radiation (similar for all organisms) and biological potassium (of which, at present, 0.0117% is ^40^K) the concentration of which varies slightly between species. For species with potassium concentrations similar to humans these produce about 0.66 mSv y^−1^ or about 60% of the radiation exposure to which terrestrial organisms are exposed.

As organisms began to colonize the land their exposure to gamma and neutron radiation increased by a factor of about 50% because of exposure to geologic sources of radiation and the loss of attenuation of cosmic radiation by the water column.

## Radiogenic DNA damage rates in the past

Based on the ratio of ^13^C :^12^C in putative “chemical fossils” the first living organisms probably appeared about 3.8 billion years ago (Ga) evolving from single-cell prokaryotes into bacterial and algal mats, eukaryotes and multi-cellular life. Evidence of organisms resembling modern life first appear in the fossil record at least 538–502 Ma. Because of the natural radioactive decay of primordial radionuclides, life on the earliest Earth likely experienced environmental radiation dose rates that were nearly an order of magnitude higher than those experienced today. Exposure likely dropped relatively slowly driven primarily by the decay of biologically incorporated ^40^K. When vertebrate life emerged to terrestrial environments the dose rate from penetrating radiations were about 15–20% higher than today [3].

Earth’s earliest atmosphere lacked free oxygen until about 2.4–2.0 Ga in what is called the Great Oxidation Event (GOE). Oxygen levels rose slowly at first and more sharply beginning about 800 Ma, peaking at nearly 35 percent in the Carboniferous Period (between 400 and 350 Ma) before dropping to roughly the current 21 percent. Oxygen levels are important. They give rise to the ozone layer that blocks ionizing ultraviolet radiations. Oxygen also enhances DNA damage caused by ionizing radiations [7].

In summary, the first organisms to adopt a partially or fully terrestrial lifestyle lost the shielding provided by the water column at a time when environmental radiation levels, atmospheric oxygen concentrations and, consequently, rates of radiogenic DNA damage were substantially higher than today.

## Radiation attenuation by bone and tissue

Attenuation of gamma radiations can be calculated using software such as XCOM (National Institute of Standards and Technology, https://www.nist.gov/pml/xcom-photon-cross-sections-database) or MicroShield^®^ (Grove Software, Lynchburg VA, USA). Using attenuation coefficients for bone and tissue generated by XCOM we calculated the reduction in radiation exposure rates to haematopoietic cells in the bone marrow cavity (a detailed explanation of these is available from the authours). These calculations for various thicknesses of bone and tissue (from 0 to 10 mm of bone and from 0 to 20 mm of tissue) indicate the natural background radiation spectrum is attenuated by up to 40% with some combinations attenuating the background gamma radiation spectrum by 15–25 percent. It is worth noting that the median thickness of the human ilium has a maximum value of 42 mm [8].

Bone and soft tissue attenuate radiation by absorbing and scattering incident photons. With a density of slightly >3 gm cm^−3^ bone is an effective shield against gamma and x-ray radiations with soft tissues providing additional attenuation of gamma and neutron radiations. In adult humans about one-half of the contents of bone marrow-bearing cavities such as the ilium are fat cells attenuating gamma and neutron exposure further still.

## Hypothesis

We suggest translocation of haematopoietic cells to the bone marrow cavity is an evolutionary adaptation to increased environmental radiation exposures which occurred when aquatic organisms adopted a terrestrial lifestyle in an era of unusually high atmospheric oxygen concentrations. Our hypotheses is supported by observations such as translocation of haematopoiesis from the mesonephros to the bones in aquatic compared with terrestrial frogs. Amphibious frogs (including the tadpole form) have a mixture of mesonephros and bone marrow cavity haematopoiesis [9–11]. One may wonder why germ cells are not similarly resident within bone given their radiation sensitivity. An increase in radiation-induced mutations in these cells may provide a small evolutionary advantage by increasing genetic variability. Although we know of no way to definitively prove or disprove our hypothesis we welcome discussion.

## Summary and conclusions

Unlike aquatic organisms whose haematopoietic cells are largely shielded from environmental radiations by the water those of adult terrestrials are predominately in the bone marrow cavity. This translocation coincides with time when fully terrestrial organisms first appeared. Hematopoietic cells are among the most sensitive to damage from exposure to ionizing radiations and when vertebrates moved from sea to land radiogenic DNA damage rates were >20 percent higher than now. Placing haematopoietic stem cells in the bone marrow cavity reduces radiation exposure by 10–40 percent. Consequently, it seems reasonable to hypothesize this translocation might be driven by the radiation shielding provided by bone and overlying tissues. We hypothesize an evolutionary advantage to the terrestrial organisms which translocated hematopoiesis to within the bone marrow cavity by preventing radiation-induced mutations in long-lived haematopoietic stem cells.

## Disclaimer

Any views expressed in this article are those of the authors and do not represent the views of the Department of Homeland Security nor US Government.
